# Research Ethics Training in Peru: A Case Study

**DOI:** 10.1371/journal.pone.0003274

**Published:** 2008-09-26

**Authors:** A. Roxana Lescano, David L. Blazes, Silvia M. Montano, Zoe Moran, Cesar Naquira, Edwin Ramirez, Reidar Lie, Gregory J. Martin, Andres G. Lescano, Joseph R. Zunt

**Affiliations:** 1 United States Naval Medical Research Center Detachment, Lima, Peru; 2 Peruvian Ministry of Health, Instituto Nacional de Salud, Lima, Peru; 3 Hospital Nacional Dos de Mayo, Lima, Peru; 4 Department of Clinical Bioethics, National Institutes of Health, University of Washington, Seattle, Washington, United States of America; 5 Departments of Neurology, Global Health and Medicine, University of Washington, Seattle, Washington, United States of America; Instituto de Medicina Tropical Alexander Von Humboldt, Peru

## Abstract

With the rapidly increasing number of health care professionals seeking international research experience, comes an urgent need for enhanced capacity of host country institutional review boards (IRB) to review research proposals and ensure research activities are both ethical and relevant to the host country customs and needs. A successful combination of distance learning, interactive courses and expert course instructors has been applied in Peru since 2004 through collaborations between the U.S. Naval Medical Research Center Detachment, the University of Washington and the Department of Clinical Bioethics of the National Institutes of Health to provide training in ethical conduct of research to IRB members and researchers from Peru and other Latin American countries. All training activities were conducted under the auspices of the Peruvian National Institute of Health (INS), Ministry of Health. To date, 927 people from 12 different Latin American countries have participated in several of these training activities. In this article we describe our training model.

## Introduction

Interest in global health research and training is rapidly increasing, especially among medical students. [1] As more U.S. medical centers strive to provide international research and clinical experiences, the requirement for certified international institutional review boards (IRB) in the research arena has also increased. In addition, large pharmaceutical companies, conduct 29% to 70% of their clinical trials in developing countries, although clear figures are unavailable. [2] Transjurisdictional research requires the approval of both originating country and the country of operation. IRBs or Ethics Committees in developed countries often have little grasp of the conditions for ethical review in other and particularly developing countries. Additionally, there is concern in developed countries that research, particularly industry sponsored, is migrating ‘off shore’ due to lower costs, but more particularly, less burdensome regulatory environments.

To ensure international research protects the rights and welfare of human subjects, the Office of Human Research Protection (OHRP) of the U.S. Health and Human Services requires all federally-sponsored research conducted on human subjects at international sites have approval by an IRB holding an OHRP Federal wide Assurance (FWA).

Each institution with a FWA is responsible for ensuring investigators conducting HHS-supported human subjects research understand and act in accordance with the requirements of the HHS regulations for the protection of human subjects. Therefore, as stated in the Terms of the FWA, OHRP strongly recommends that institutions and their designated IRBs establish training and oversight mechanisms to ensure investigators maintain continuing knowledge of, and comply with relevant ethical principles and federal regulations, written IRB procedures, OHRP guidance, state, local laws and international laws, and institutional policies for the protection of human subjects. In addition, OHRP recommends investigators complete institutional educational training before conducting human subjects research; in some instances, such as for the National Institute of Health, training is mandatory for all key personnel conducting NIH-sponsored human subjects research. In addition to the ethical aspects of clinical research, other areas of equal importance include requirements for authorship and dissemination of research results. One of the conclusions of the Ethics of Research Related to Healthcare in the Developing Countries, specifies that ‘there is an urgent need to further education and training to ensure that those [researchers] in developing countries are able to discuss ethical issues effectively with external sponsors and others and to have mechanisms in place to deal with issues that arise. [3]

Strengthening bioethics training for both young and seasoned researchers in Latin America is a vital need, particularly as foreign-funded research conducted in this part of the world continues to increase. Training resources in human research protection are available over the internet and several of them are free of charge. The Collaborative Institutional Training Initiative (CITI) modules offer one of the most complete programs, ranging from Basic Aspects of Human Subject Protection, Good Clinical Practices, Responsible Conduct of Research among many others. Many of their modules have been translated to Spanish and adapted to local practices. The Collaborative Institutional Training Initiative (CITI) modules were first introduced in Peru at the 2007 Conference in Lima and were very well received by the audience. Between May and August 2007, 804 individuals had requested access to the Spanish version of the Basic course module, 90% of whom were Peruvian, The Office of Human Research Protection (OHRP), the National Cancer Institute (NCI), and Family Health International (FHI) also have human subject protection training modules geared to investigators and/or IRB members.. These resources are especially useful to existing research programs in the United States, but may not be as relevant for scientific communities in the developing world in the absence of structured institutional Human Research Protection Programs and lack the one-on-one approach. The Helsinki Declaration issued by the World Medical Association and the International Ethical Guidelines for Biomedical Research published by the International Organizations of Medical Sciences (CIOMS) are essential reference documents for the IRB community, as well, and are discussed fully in several of these training events.

Although there is general agreement among investigators that training in ethical aspects of research is essential to conducting good and ethical studies, traditionally, clinical research grants have not provided funds for human research protection training. In addition, government and academic institutions at international sites always have limited discretionary funds for this type of training. Live courses and workshops led by experts in the field of research ethics are often prohibitively costly or available in limited geographic areas, but are especially valuable for encouraging one-on-one interaction with other investigators and opportunities for trainees to learn from case-based discussions or clarify areas of uncertainty–which are common in international biomedical ethics.

Recently international and national agencies such as the World Health Organization, the European Commission, the US National Institutes of Health, and the Wellcome Trust have shown an interest in addressing this concern and have funded initiatives for training programs and capacity building regional and national workshops. Here we will report on our experience in developing a series of live courses and workshops that could provide useful information for these newly developed programs.

## Materials and Methods

### The Training Model

The US Naval Medical Research Center Detachment Peru (NMRCD-Peru) is one of five overseas US military infectious diseases laboratories and the only one located in the Americas. The central geographic location in Peru has made it an easy-to-access country for South American colleagues who desire to participate in training activities. Through collaboration with the University of Washington, the NIH Department of Clinical Bioethics, the Peruvian National Institute of Health, the Peruvian Institutional Review Board Network and local Peruvian universities, NMRCD became the center for bioethics research training for participants from across Latin America from 2004 through 2007. Although universities provide the ideal location and resources for training activities, good networking, the support of domestic and international government and private institutions and a strong commitment from the Peruvian IRB Network made it possible for a series of training efforts to be provided by NMRCD in Peru.

The training model followed by NMRCD combined distance learning, interactive teaching and high level expert teaching in workshops, courses, conferences, webminars and videoconferences as the key element for success. Courses and conferences involved didactic sessions and mock IRB discussions conducted by experts from the U.S, and from Peru to provide a more relevant approach. Topics included conducting ethical research, informed consent, placebo versus standard of care, research with children, authorship, feedback to research subjects, repository and tissue sample banks and international collaborative research. Each participant completed a test at the end of the event and received a certificate of attendance. Participants included IRB Chairs and members, researchers, persons directly involved in clinical research and professionals directing offices in academic or research organizations. All courses and conferences were co-sponsored by the Peruvian Medical Board Association and local Universities and continuing medical education credits were awarded to participants who completed the post-test.

The most popular training courses have been the Conferences on Ethical Aspects of International Collaborative Research held in Lima, and Iquitos, a city in the Peruvian Amazon region; and the Conference in Ethics in Collaborative International Research: Practical Issues and Constructive Tools for Latin American Research Teams held in Lima and Arequipa, a city in the Southern Andes. The satellite conferences gathered approximately 80 students each, while the Lima-based conferences had nearly 200 participants each. The lectures presented various topics of crucial importance to ethics in research, such as coercion, undue inducement, exploitation, informed consent, research with vulnerable populations, placebo versus standard of care, research with children, criteria for authorship, feedback to the research subjects, repository and tissue sample banks and international collaborative research. To better illustrate topics and make them more relevant to South America, faculty incorporated results and case-studies from research conducted in Peru and other parts of the world. Faculty members were well-published speakers from the U.S. OHRP and NIH Department of Clinical Bioethics, the Universities of Washington and Texas and Peruvian academic and regulatory entities and IRBs. The events were organized in collaboration with Peruvian organizations, such as the Universidad Peruana Cayetano Heredia, Universidad Nacional Mayor de San Marcos, the Peruvian Medical Association, the Peruvian Ministry of Health, the Peruvian National Institute of Health. The University of Washington provided funding and coordination for the Ethics in Research Courses conducted in Lima and Iquitos in 2005 and the Lima Conference on Ethical Aspects of International Collaborative Research and course in Arequipa in the 2007. The U.S. NIH Department of Clinical Bioethics played a crucial role in providing training through the 2004–2007 video conference course entitled NIH Ethics and Regulatory Practices in Clinical Research, the three Latin American Conferences on Ethical Aspects of International Collaborative Research held in Lima in 2005, 2006 and 2007, and the Ethics in Research course held in Iquitos in 2006. The videoconference course includes 7 sessions and is simulcast to multiple sites throughout the U.S., Peru, Mexico and Puerto Rico.

**Figure 1:**
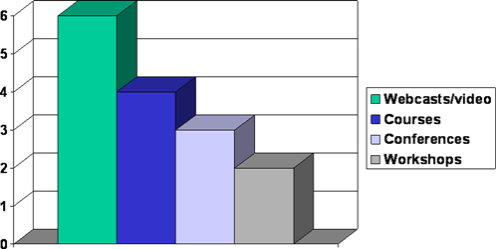
Types of training events held in Peru, 2004–2007.

Since 2004, other training activities have included: webcast broadcasting on ethics in international clinical trials and the Anniversary of the Belmont Report; and two workshops for IRB Administrators (Figure 1).

## Results

**Figure 2:**
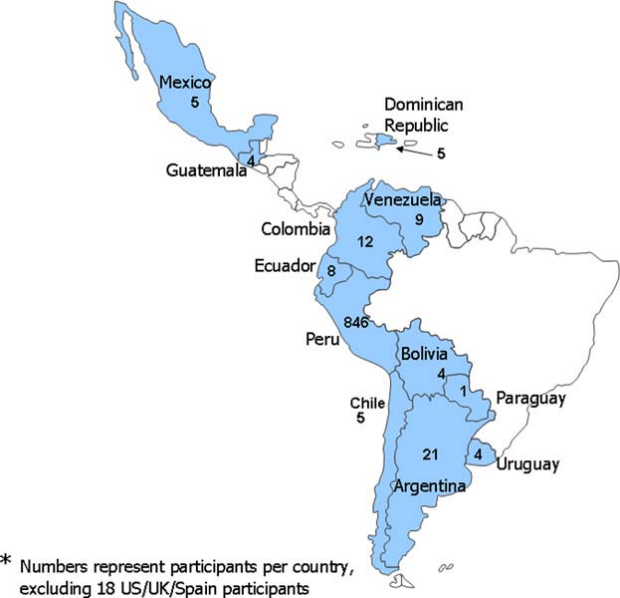
Courses and participants, 2004–2007.

A total of 927 (258 of whom were IRB members) from 12 different countries in the Americas participated in training courses between 2004 and 2007 (Figure 2). Of the 927 participants, 836 were Peruvian and 510 of these were Peruvian MoH staff. Forty-nine percent (49%) of the participants were women. Suggestions received from the students encouraged the organization of more courses and post-test results demonstrated recognition of basic concepts of ethics in research.

A total of 137 of the 804 Peruvians who registered to take the CITI online modules completed the CITI course entitled Basic Aspects in the Protection of Human Research Subjects within the first 10 months.

**Figure 3:**
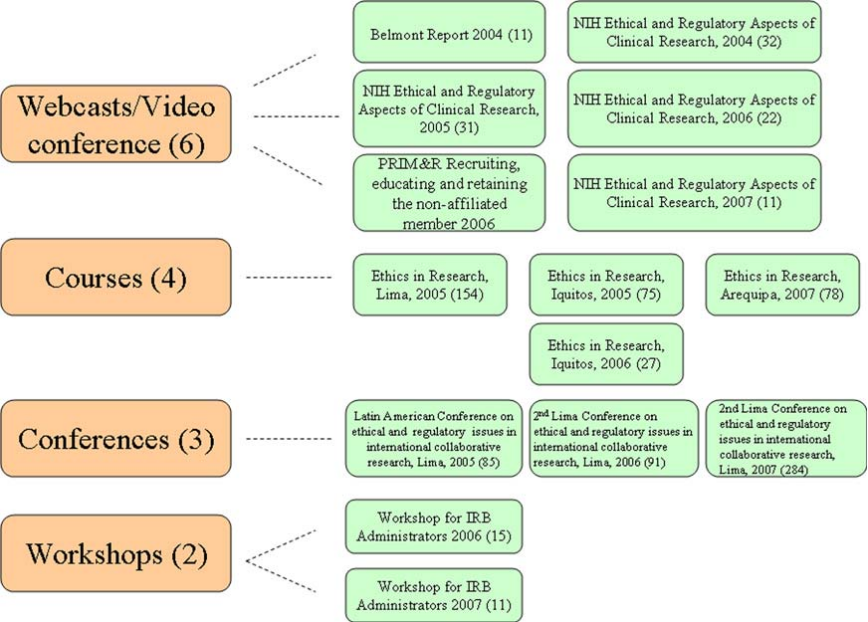
Training activities conducted.

The NIH Ethics and Regulatory Practices in Clinical Research video conference course has been so successful in Peru that since 2005, two additional sites in Lima were registered directly with the NIH to broadcast the course sessions from their own facilities. Participants in the videoconference series maintained a 70–80% attendance rate and anonymous surveys showed high satisfaction with the overall course (Figure 3). A total of 95 Peruvians received NIH certificates of participation from 2004 through 2007.

## Discussion

The developing world needs both, bioethics training and IRB capacity reinforcement to ensure research conducted in each country is compliant with international standards, while at the same time, sensitive to the needs of the local populations. Our collaborative bioethics conferences co-hosted by Peruvian academic and governmental organizations, U.S. NMRCD, and U.S. based government and academic institutions provided a unique opportunity for fostering adherence to ethical standards of research in this region. In Peru, this partnership has been extremely fruitful, with the participation of over 927 professionals from 12 different Latin American countries.

We believe inclusion of expert speakers, a diverse curriculum and the investment and commitment of local partners made our conferences successful. Involvement of Peruvian members in both the presentations and mock IRBs promoted the inclusion of topics relevant to the developing world and fostered greater understanding between investigators and IRB members from developing and developed countries. Although conference training lacked an applied, practical component, many of the participants had extensive experience already and benefited from reinforcement of theoretical concepts and examples from research conducted in other parts of the world. This training model can be easily reproducible by other developing world countries.

Training courses on bioethics are essential for encouraging acknowledgement and understanding of the importance of ethical conduct among persons conducting clinical research in the developing world. In addition, these courses strengthen the capabilities of IRB members and encourage better functioning of existing IRBs and the creation of new ones. Our courses and conferences were perceived as very useful by the Latin American scientific community, with a-growing number of attendees registering for these events and requesting additional training opportunities. We believe the next step is to target more advanced individuals, such as IRB chairs and members and develop intensive site evaluations to assist with setting up systems for record-keeping, IRB activity monitoring, tracking modifications and continual renewals, as well as encouraging local hosting of additional training courses.

Although our courses and conferences have received positive evaluations from the participants, we recognize that such evaluations provide limited evidence of the usefulness of the training program. Ideally, we would like to know if the training program contributes to better research ethics review and ultimately to better protection of research participants. We are not aware of any formal evaluations of training programs using such criteria, and it would be almost impossible to do such an evaluation in a rigorous manner. One could, however, measure the level of knowledge and understanding of ethical principles and human subjects regulations before and after a series of training courses. Again, there are no standardized instruments for such evaluations available right now. Given the increasing interest in funding training workshops by international agencies, we believe that the development of such an instrument should be of high priority.
